# Effect of 25% in-office hydrogen peroxide bleaching on flexural strength of resin-based composites: an in vitro study

**DOI:** 10.1186/s12903-026-08240-7

**Published:** 2026-04-02

**Authors:** Vani Grover, Shashi Rashmi Acharya, Akshatha Chatra, Arun Mayya

**Affiliations:** Department of Conservative Dentistry and Endodontics, Manipal College of Dental Sciences, Manipal Academy of Higher Education, Manipal, 576104 Karnataka India

**Keywords:** Resin-based composites, Bleaching, Hydrogen peroxide, Flexural strength, Giomer, Microhybrid composite, Universal composite

## Abstract

**Background:**

Resin-based composites (RBCs) are commonly used in restorative dentistry, but bleaching agents may compromise their mechanical integrity. The effect of in-office bleaching with 25% hydrogen peroxide (HP) on the flexural strength (FS) of contemporary RBCs remains unclear.

**Materials and methods:**

An in vitro study was conducted using three resin composites: Beautifil II (giomer), Spectrum (microhybrid), and Omnichroma (universal). Seventy-two bar-shaped specimens (25 × 2 × 2 mm) were allocated into six groups (*n* = 12 each). Three groups served as unbleached controls representing giomer, microhybrid, and single-shade universal resin composites stored in distilled water. The other three groups were the corresponding bleaching subgroups, treated with 25% hydrogen peroxide gel activated in three 15-minute light-assisted cycles (total 45 min). Bleaching was performed with 25% HP gel (Philips Zoom) applied in three 15-min cycles with light activation. The FS was measured by three-point bending according to ISO 4049. The data were analyzed using two-way ANOVA and Tukey’s post hoc tests (*p* < 0.05).

**Results:**

Bleaching with 25% HP had no significant effect on FS across materials (*p* = 0.618). The resin composite type significantly influenced the FS (*p* < 0.001). Spectrum exhibited higher FS values than Beautifil II and Omnichroma, which were statistically similar.

**Conclusion:**

In-office bleaching with 25% HP did not reduce the FS of the tested RBCs. The material composition had a greater impact on FS than bleaching, with the microhybrid resin composite (Spectrum) performing better than the giomer and universal resin composites.

## Introduction

Resin-based composites (RBCs) are widely used in conservative and esthetic dentistry because they combine favorable mechanical properties with excellent optical performance [1]. They consist of a dimethacrylate resin matrix reinforced with inorganic fillers, which typically comprises more than 50 wt%. The degree of conversion and cross-link density of the resin, together with the filler type and distribution, influence the flexural strength (FS), modulus, and wear resistance [2–5]. Contemporary RBCs include microhybrids, giomers, and universal shade formulations. Microhybrids blend micron- and nanosized fillers for strength and polishability, giomers incorporate surface-prereacted glass (S-PRG) particles with fluoride release but exhibit lower strength [6–9]. Universal resin composites such as Omnichroma rely on uniform supra-nanofillers to achieve structural coloration, and their mechanical properties have been reported to be comparable to those of conventional resin composites [10]. FS testing, particularly the ISO 4049 three-point bending method, is commonly employed to assess the ability of RBCs to withstand occlusal loads [11, 12].

Dental restorations are subject to chemical and mechanical challenges that can degrade the resin and filler matrix interface [13]. Bleaching agents such as hydrogen peroxide (HP) and carbamide peroxide (CP) are frequently used to meet esthetic demands. These agents diffuse through enamel and dentin, generating reactive oxygen species that oxidize organic pigments within dental tissues [14]. However, the same radicals may compromise the resin matrix and filler bonding [15]. The literature reports mixed results: low-concentration at-home bleaching (≈ 10% CP) often has no significant effect on FS, whereas higher concentrations (15–40%) have been associated with decreased FS and microhardness in some resin composites and hybrid ceramics [16–19]. Bleaching has also been shown to increase surface roughness, which could predispose restorations to staining and wear [20]. Clinically, in-office and at-home bleaching are increasingly performed in patients who already have RBC restorations, raising concerns about possible weakening of existing restorations and the longevity of aesthetic results.

The materials selected represent distinct categories: a giomer (Beautifil II) with fluoride-releasing S-PRG fillers, a conventional microhybrid resin composite (Spectrum) with established clinical use, and a universal single-shade resin composite (Omnichroma) based on supra-nanofillers for structural coloration. This selection allowed assessment of whether differences in filler technology influence susceptibility to bleaching and provided a representative comparison of contemporary RBC classes. Such comparative evaluation is clinically meaningful because these resin composite classes differ in resin chemistry and filler phase architecture, which govern stress distribution and oxidative radical diffusion behavior in high-concentration light-activated systems such as Philips Zoom Advanced Power Light.

Although numerous studies have investigated the effects of bleaching agents on resin composites across broad concentration ranges or focused primarily on surface-level properties, the effect of a clinically relevant 25% in-office hydrogen peroxide regimen on bulk flexural strength has been less clearly isolated, particularly when evaluated using a strictly standardized ISO 4049 three-point bending protocol across resin composites with distinct filler technologies. The novelty of the present study lies in its controlled evaluation of a single, commonly used light-activated 25% HP protocol on the bulk flexural strength of giomer, microhybrid, and universal resin composites, thereby allowing material-dependent responses to be compared under uniform testing conditions.

Therefore, this study evaluated the influence of a 25% HP in-office bleaching regimen on the FS of Beautifil II (giomer), Spectrum (microhybrid), and Omnichroma (universal) using the ISO 4049 three-point bending test. Two null hypotheses were tested: (1) that 25% hydrogen peroxide bleaching would not significantly affect the flexural strength of RBCs, and (2) that no difference in flexural strength would exist among the tested resin composites.

## Materials and methods

This in vitro study was approved by the institutional ethics committee. Three light-cured RBCs were selected: Beautifil II (nanohybrid; Shofu Inc., Kyoto, Japan; shade A2), Spectrum (microhybrid; Dentsply Sirona, Charlotte, NC, USA; shade A2) and Omnichroma (supra-nanofilled universal shade; Tokuyama Dental Corp., Tokyo, Japan). The bleaching agent was a light-activated in-office gel containing 25% hydrogen peroxide (Philips Zoom; Discus Dental LLC, Ontario, CA, USA). Table 1 summarizes the compositions of the tested materials.

**Table 1:** Composition of the tested RBCs

Material	Type	Manufacturer (City, Country)	Shade	Resin Matrix	Filler Type/Content	Special Features
Beautifil II	Giomer (nanohybrid)	Shofu Inc., Kyoto, Japan	A2	Bis-GMA, TEGDMA	Surface-prereacted glass (S-PRG) fillers + micro/nanofillers; ~83 wt%	Fluoride release from S-PRG phase
Spectrum	Microhybrid	Dentsply Sirona, Konstanz, Germany	A2	Bis-GMA, UDMA, TEGDMA	Barium glass fillers; ~78 wt%	Well-documented clinical performance
Omnichroma	Universal single-shade (supra-nanofilled)	Tokuyama Dental Corp., Tokyo, Japan	Universal	UDMA, TEGDMA	Uniform supra-nanofillers (260 nm spherical)	Structural coloration enabling single-shade blending
Philips Zoom In-Office Bleaching Gel	In-office bleaching agent	Discus Dental LLC, Ontario, Canada	Not applicable	Not applicable	Not applicable	25% hydrogen peroxide; light-activated in-office bleaching system

### Sample size calculation

Based on the results of a previous study [21], a priori sample size calculation was performed using G*Power software (version 3.1.9.7). For a two-way ANOVA (3 resin composite types × 2 bleaching conditions; six groups), assuming an effect size of Cohen’s f = 0.40, α = 0.05, and power (1–β) = 0.80, the minimum total sample required was 64. To ensure equal allocation across groups, we used 72 specimens (*n* = 12 per group).

### Specimen preparation

A total of 72 bar-shaped specimens (25 × 2 × 2 mm; *n* = 12 per subgroup) were fabricated using a stainless-steel split mold according to ISO 4049 specifications. The mold was placed between glass slides and secured on a glass slab. The resin composite was packed incrementally and covered with a second glass slide to remove excess material and standardize the surface. Polymerization was performed with an light-emitting diode (LED) curing unit (Ortholux Luminous, 3 M UNITEK, Monrovia, CA, USA) at 1000 mW/cm² for 40 s, followed by an additional 20 s of exposure on each surface after removal from the mold. The light tip was held at 90° to the specimen surface at a distance of ≤ 1 mm. All specimens were examined under 3.5× magnification to exclude voids and defects and then stored in distilled water at 37 (±) 1 °C for one week before testing.

### Grouping and bleaching protocol

Specimens were randomly allocated into six groups (*n* = 12 each): three control subgroups (unbleached) and three experimental subgroups (bleached). Bleaching was carried out using the Philips Zoom system. A 1–2 mm thick layer of bleaching gel was applied to the specimen surface and activated with Zoom Advanced Power Light for 15 min. The procedure was repeated three times, with fresh gel for each cycle, for a total exposure time of 45 min. Control specimens were stored in distilled water at 37 (±) 1 °C for the same duration. After treatment, specimens were rinsed, air-dried, and stored in distilled water for 24 h until testing.

### Flexural strength testing

Flexural strength was measured with a universal testing machine (Instron 3366, Instron Corp., Norwood, MA, USA) in three-point bending mode with a 20 mm support span and a crosshead speed of 1 mm/min at room temperature. The three-point bending methodology and test parameters were adopted from previously published protocols for resin composite flexural strength assessment [11]. The width and thickness were measured with a digital caliper (± 0.01 mm) at three points, and the mean values were used to calculate the flexural strength (σ) according to the following formula: σ = 3FL/(2bd²), where *F* is the maximum load at fracture (N), *L* is the support span (mm), and *b* and *d* are the specimen width and thickness (mm), respectively.

### Statistical analysis

Data were analyzed using Jamovi software (version 2.3.24; Sydney, Australia). Normality was tested using Shapiro–Wilk test, and homogeneity of variance with Levene’s test. Two-way ANOVA was performed to evaluate the effects of resin composite type and bleaching, as well as their interaction, on flexural strength. Tukey’s post hoc test was applied for pairwise comparisons. Statistical significance was set at *p* < 0.05.

## Results

**Fig. 1:**
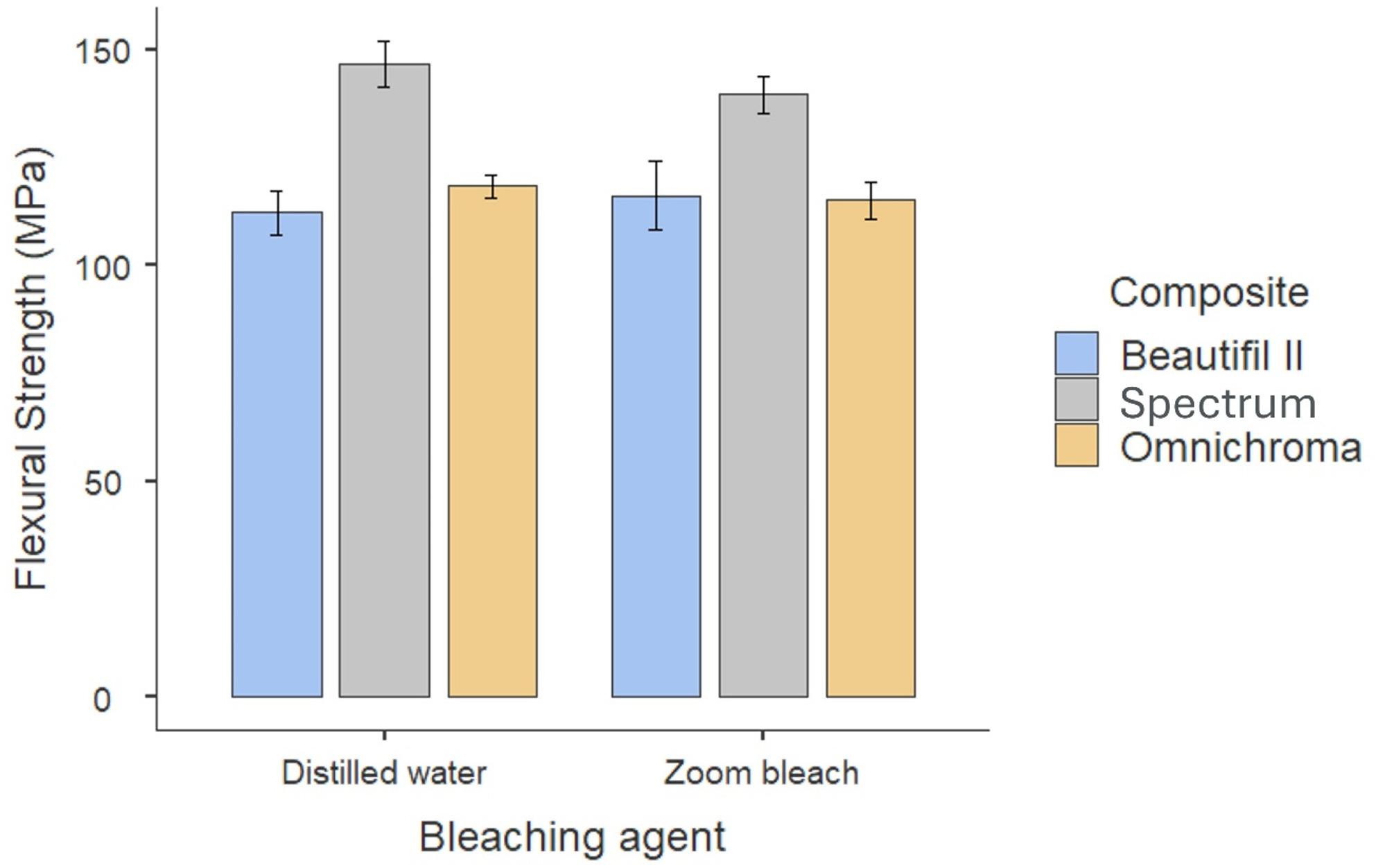
Bar-plot shows the 95% CI of the mean flexural strength values across different groups

The descriptive statistics are shown in Table 2. The FS values (MPa) were as follows: Beautifil II control 112.15 ± 18.09, Beautifil II bleached 116.15 ± 27.97, Spectrum control 146.75 ± 18.68, Spectrum bleached 139.55 ± 15.24, Omnichroma control 118.30 ± 8.96, and Omnichroma bleached 115.04 ± 15.04. Spectrum exhibited the highest flexural strength (FS) in both the control and bleached conditions, whereas Beautifil II and Omnichroma presented lower and comparable values (Table 2; Fig. 1).

**Table 2:** Descriptive summary of flexural strength (MPa) across different resin composites and bleaching agents

	Bleaching agent					
	Distilled water		Zoom bleach		Total	
Composite	*n*	Mean±SD	*n*	Mean±SD	*n*	Mean±SD
Beautifil II	12	112.15±18.09	12	116.15±27.97	24	114.15±23.12
Spectrum	12	146.75±18.68	12	139.55±15.24	24	143.15±17.08
Omnichroma	12	118.30±8.96	12	115.04±15.04	24	116.67±12.22
Total	36	125.73±21.71	36	123.58±22.83		

Two-way ANOVA (bleaching × resin composite type) revealed no interaction effect between bleaching and resin composite type (F₂,₆₆ = 0.58; *p* = 0.561). Bleaching had no main effect on FS (F₁,₆₆ = 0.25; *p* = 0.618). Resin composite type, however, had a significant main effect (F₂,₆₆ = 18.61; *p* < 0.001). Thus, the material performance was consistent across the bleaching treatments. Tukey’s post hoc comparisons showed Spectrum had significantly greater FS than did Beautifil II (mean difference = 29.00 MPa, *p* < 0.001) and Omnichroma (mean difference = 26.48 MPa, *p* < 0.001). No significant difference was detected between Omnichroma and Beautifil II (mean difference = 2.52 MPa, *p* = 0.882). 

## Discussion

This in vitro study evaluated the effect of a clinically used 25% light-activated hydrogen peroxide bleaching protocol on the bulk flexural strength of standardized resin composite specimens. The selected materials represented distinct filler–resin architectures, including S-PRG–based giomer, microhybrid barium glass–reinforced, and single-shade supra-nanofilled structural coloration systems, enabling a controlled inter-category comparison. In this context, the present findings demonstrate that isolating a single, clinically used in-office bleaching concentration and evaluating bulk mechanical behavior using a standardized ISO 4049 three-point bending protocol allows material-dependent responses to be interpreted without confounding effects of multiple concentrations or surface-limited analyses. Accordingly, the first null hypothesis that 25% hydrogen peroxide bleaching would not affect flexural strength was accepted, whereas the second null hypothesis that no difference would exist among resin composite types was rejected.

The results of the present study align with reports that lower-concentration at-home bleaching regimens (≈ 10% carbamide peroxide) do not reduce the FS of microhybrid/nanohybrid resin composites [16]. Conversely, some studies have reported FS reductions after bleaching, depending on the concentration, regimen and material. Notably, 15% CP reduced the FS of certain RBCs but had a minimal effect on hybrid ceramics. In contrast, 40% HP significantly decreased the FS in hybrid ceramics, and similar reductions have been reported with CP concentrations between 10 and 35% [19, 21, 22]. These findings underscore that bleaching effects are material specific; the results from hybrid ceramics should not be directly extrapolated to RBCs. These discrepancies reflect differences in peroxide concentration, application protocol (time, refresh, and activation), and composite architecture (matrix chemistry and filler loading/size), which together govern radical diffusion and potential degradation at the resin–filler interface. In the present protocol, gel was applied in three 15-min cycles with light activation (total 45 min), which may have limited radical penetration into the bulk and preserved FS under the tested conditions. This observation is consistent with peroxide radical diffusion models, which show that short, cyclic light-activated applications predominantly produce oxidative changes up to shallow subsurface depths and may not generate sufficient bulk polymer network compromise to reduce flexural strength. The gel refresh strategy used in the present protocol potentially further limited sustained oxygen radical concentration gradients in the specimen core, preserving stress-bearing integrity under bending loads.

Material-wise, Spectrum exhibited a greater FS than Beautifil II and Omnichroma did, which is consistent with expectations for microhybrids that combine micron-scale glass and nanofillers to resist bending, which is supported by effective matrix–filler coupling and a high proportion of reinforcing glass fillers [23]. The lower FS of Beautifil II could be associated with the presence of S‑PRG fillers; although they provide fluoride release and recharging ability, the ionomer phase may act as a defect under bending stress [24].

Previous investigations have also highlighted material-dependent differences in flexural strength. Llancari-Alonzo et al. [24] reported that Beautifil II (giomer) released fluoride and exhibited moderate FS values among ion-releasing restorative materials, an outcome attributed to the presence of an S-PRG phase that may compromise filler–matrix coupling. Similarly, Ren et al. [23] reported that dense microhybrid resin composites outperformed nanohybrid and giomer-based materials in FS, reflecting the reinforcing effect of micron-scale glass fillers combined with nanofillers. With respect to universal resin composites, Oliveira et al. [10] reported that single-shade systems, including Omnichroma, achieved FS values comparable to those of a conventional nanohybrid, whereas Alharbi et al. [25] reported that Omnichroma exhibited a lower FS than a nanohybrid control, although still within a clinically acceptable range. Collectively, these results support the present findings that Spectrum (microhybrid) had a greater FS than did Beautifil II and Omnichroma, while the latter two had comparable values.

Beautifil II (giomer) contains an S-PRG phase that may alter filler–matrix coupling, whereas Omnichroma relies on uniform supra-nanofillers to achieve structural coloration; either feature could contribute to the comparable but lower FS observed relative to Spectrum [24, 26]. Our results align with previous findings that filler type and polymer network structure influence FS more than bleaching treatment does [16, 27, 28].

The absence of a bleaching effect on FS indicates that, under the tested regimen, the bulk resistance to bending was maintained. Between-material differences likely reflect a combination of matrix chemistry and filler characteristics, but the present study did not quantify these parameters [29]. Although FS was maintained, bleaching effects on surface and chemical properties cannot be inferred from this study, as parameters such as surface roughness, hardness, or matrix chemical changes were not evaluated here. Previous literature has documented peroxide-induced surface changes in RBCs, including increased surface roughness and reduced microhardness across different resin composite categories [30, 31]. Popescu et al. [32] reported that bleaching increased the surface roughness of several resin composites regardless of composition, and Vaizoğlu et al. [33] reported similar effects on universal resin composites. These studies reinforce that peroxide influence, when reported, is predominantly surface-limited, whereas the current investigation evaluates bulk flexural strength behavior only.

The present study has limitations inherent to in vitro experiments. Specimens were stored in distilled water and not subjected to thermocycling or mechanical loading; dynamic oral conditions such as pH fluctuations, temperature changes and mastication may influence bleaching penetration and resin degradation. The sample size, although consistent with previous studies, may limit the generalisability of the findings. Only three resin composites and one bleaching protocol were evaluated; other formulations, such as bulk‑fill, flowable, or fiber‑reinforced composites, may respond differently. Future research should examine the long‑term effects of bleaching under simulated clinical conditions, compare in‑office and at‑home protocols across various resin composite types and explore strategies to mitigate bleaching‑induced surface changes. Additionally, future work may incorporate surface roughness, microhardness, color stability assessment (e.g., using the Vickers microhardness test or CIELab colorimetric analysis), and chemical profiling via FTIR or Raman spectroscopy to provide a fuller structure–property evaluation of peroxide susceptibility.

In summary, 25% HP did not reduce the FS of the tested resin composites under the conditions used. Clinicians should remain aware of potential surface changes and consider finishing/repolishing after bleaching; careful control of the bleaching concentration, activation, and duration may help preserve restoration performance.

## Conclusion

Within the limitations of this in vitro study, in‑office bleaching with 25% HP applied in three 15-min cycles did not significantly affect the flexural strength of the Beautifil II, Spectrum or Omnichroma resin composites. The resin composite type influenced the flexural strength, with Spectrum (microhybrid) showing higher values than those of the giomer and universal materials. Further research is required to evaluate untested surface and chemical parameters using advanced analytical tools.

## Data Availability

The datasets generated and analyzed during the current study are available from the corresponding author on reasonable request.

## Abbreviations

RBC: Resin-based composites
FS: Flexural strength
HP: Hydrogen peroxide
CP: Carbamide peroxide
LED: Light-emitting diode
S-PRG: Surface-prereacted glass
ISO: International Organization for Standardization
Bis-GMA: Bisphenol A glycidyl methacrylate
TEGDMA: Triethylene glycol dimethacrylate
UDMA: Urethane dimethacrylate
